# Singular Effect of a Blow on the Occupit

**Published:** 1856-10

**Authors:** 


ARTICLE IV.—*Singular Effect of a Blow on the Occiput.* By the EDITOR.

Mr. F., a native of Ireland, aged about 22 years, previously in good health, while sitting on his sewing bench at work as a tailor, received a severe blow with a press-board directly on the most prominent part of the occiput.

The blow was sufficient to lacerate the scalp and produce temporary unconsciousness. From the latter he soon recovered, and, after having the wound dressed by a surgeon, he was carried to his lodgings.

The following day, June 8th, I was called to see him. I found the wound looking well, and the patient without any symptom of disease or injury, except a slight acceleration of pulse and heat of skin, with total inability to swallow either fluids or solids. If the patient made a strong effort to swallow, the liquid would no sooner reach the pharynx than it was forcibly ejected through the mouth or nostrils, or both, accompanied by severe spasmodic action of the diaphragm and great distress in the epigastrium. Finding no evidence of either fracture of the skull or compression of the brain, I simply directed cold applications to the occiput, the bowels to be moved by an enema of salt and water, and entire quietude.

The next day, I found the patient in precisely the same condition, except that the slight febrile symptoms had disappeared; the bowels had moved well, and the patient felt well, except the entire inability to swallow a drop of anything, and the spasmodic action of the diaphragm and respiratory muscles consequent upon every attempt to do so. I directed a continuance of the cold cloths to the head, and a blister to the back of the neck.

*June 11th.*—The blister drew well, but produced no change in the symptoms. The patient, when at rest, feels perfectly well, except a sense of oppression or tightness around the lower part of the chest; the skin is cool; pulse 70 per minute, soft and natural; the wound in the scalp nearly healed, and entirely destitute of inflammation. The inability to swallow was still complete, although the efforts to do so were accompanied by less spasmodic action of the diaphragm, and less distress than at first. To afford the patient some support, and, at the same time, produce some change in the condition of the nervous system, I directed a teacupful of beef-tea, containing ten drops of tinct. opii, and the twelfth of a grain of strychnine to be introduced into the rectum, each morning, noon, and night, These were continued daily, the whole being retained until completely absorbed, and they afforded the patient so much nourishment that very little emaciation ensued, although the patient remained without swallowing a drop until the 22d, fifteen days after the injury.

From the 16th to the 22d there was a gradual change: the spasmodic action which was so distressing to the patient at first, whenever an attempt was made to swallow, entirely ceased, and liquids would pass at least two inches down the œsophagus, where they would be invariably arrested and forced back. After causing the patient to make a faithful, but unsuccessful, attempt to swallow both liquids and solids, on the 22d I passed an elastic stomach tube through the œsophagus into the stomach; through it I introduced half a pint of cold water, there being neither beef-tea nor milk at hand, though I had asked them to have both ready: the tube was then withdrawn. In passing it, there seemed to be a very slight resistance about midway between the pharynx and the cardiac orifice of the stomach. It gave the patient no pain, and at no time had there been any soreness of the throat. In a few hours after the stomach tube was withdrawn, the patient succeeded in swallowing, and in a few days entirely recovered.

